# Incidence, risk factors and clinical epidemiology of melioidosis: a complex socio-ecological emerging infectious disease in the Alor Setar region of Kedah, Malaysia

**DOI:** 10.1186/1471-2334-10-302

**Published:** 2010-10-21

**Authors:** Muhammad RA Hassan, Subhada P Pani, Ng P Peng, Kirtanaa Voralu, Natesan Vijayalakshmi, Ranjith Mehanderkar, Norasmidar A Aziz, Edwin Michael

**Affiliations:** 1 Hospital Sultanah Bahiyah, Alor Setar, Kedah, Malaysia; 2 AIMST University, Bedong, Kedah, Malaysia; 3 Kolej Poly-Tech MARA, Kota Bharu, Kelantan, Malaysia; 4 Imperial College London, London, UK

## Abstract

**Background:**

Melioidosis, a severe and fatal infectious disease caused by *Burkholderia pseudomallei*, is believed to an emerging global threat. However, data on the natural history, risk factors, and geographic epidemiology of the disease are still limited.

**Methods:**

We undertook a retrospective analysis of 145 confirmed cases extracted from a hospital-based Melioidosis Registry set up from 2005 in Hospital Sultanah Bahiyah, Alor Setar, Kedah state, Malaysia, in order to provide a first description of the contemporary incidence, risk factors, and clinical epidemiology of the disease in this putatively high risk region of the country.

**Results:**

The incidence of melioidosis in Alor Setar is remarkably high at 16.35 per 100,000 population per year. The mean age of patients was 50.40 years, with infection varying nonlinearly with age. Males (75.2%; *P *< 0.0001) predominated and the majority of cases were Malays (88.9%). The overall, crude mortality rate among the study patients was 33.8%. The proportions of cases and deaths were significantly greater among patients involved in farming, forestry and fishing and the unemployed (χ^2 ^= 30.57, *P *< 0.0001). A majority of cases (62.75%) were culture positive, with mortality in these patients being 45.05%. A large proportion (83.0%) of culture positives was also bacteremic. Pneumonia accounted for 42.06% of primary diagnoses followed in importance by soft tissue abscess. In patients with pneumonia and who were culture positive, the mortality rate was as high as 65.00%. Diabetes mellitus constituted the major underlying risk factor for developing and dying from melioidosis, occurring in 57% of all diagnosed cases. The age distribution of diabetes paralleled that of melioidosis cases. There were linear associations between cases and deaths with monthly rainfall.

**Conclusions:**

Melioidosis represents a complex socio-ecological public health problem in Kedah, being strongly related with age, occupation, rainfall and predisposing chronic diseases, such as diabetes mellitus. Among cases, bacteremic patients were associated with significantly high mortality despite provision of the recommended antibacterial therapy. The burden of this disease is likely to grow in this region unless better informed interventions targeted at high-risk groups and associated diseases are urgently implemented.

## Background

Melioidosis, a severe and highly fatal infectious disease caused by *Burkholderia pseudomallei*, a gram negative soil and fresh water saprophyte, is most common in South East Asia and northern Australia [1-3]. It is being increasingly diagnosed in other tropical regions, and is now believed to represent a serious emerging global threat [1-6]. The clinical spectrum of melioidosis is complex and wide ranging, and includes latent infection, local cutaneous lesions, sub-acute pneumonia, focal organ abscess, musculo-skeletal infection, and lethal fulminant pneumonia [2,7]. The disease can cause up to 20% of all community-acquired sepsis in the tropics, including 40% of sepsis-related mortality in northern Thailand [2]. The overall mortality for primary disease can be very high - up to 50% in northeast Thailand and up to ~20% in the higher-technology setting of Northern Australia [2,3,7,8]. Disease occurs after bacterial contamination of breaks in the skin following contact with water or soil. A pneumonic form of the disease can also result from the inhalation of contaminated dust. This potential for the bacterium to cause disease after inhalation has resulted in the inclusion of this pathogen on the Centers for Disease Control list of potential biothreat agents [9,10]. Yet, despite this, the great variety of clinical presentations and difficulties with laboratory diagnosis mean that accurate information on the natural history of the disease, epidemiological risk factors, and geographic distribution both within specific regions and globally is still limited.

In Malaysia, the disease has been recorded from patients in Johor Bahru [11], Pahang state [12] and Kuala Lumpur [13] in the south, eastern and middle of the country respectively, but to date despite its proximity to Thailand and as the major rice growing region of the country, the incidence and epidemiological patterns of the disease in the northern state of Kedah is unknown. Here, we analyze retrospective data from a Melioidosis Registry maintained at Hospital Sultanah Bahiyah (HSB) in the state capital to record clinical, treatment and outcome information from all local cases which were culture and/or serologically positive for the disease between the years 2005 and 2008, in order to present a first evaluation of the contemporary incidence, epidemiology and outcomes of the disease in this putatively risky region of the country.

## Methods

### Study population

This is a hospital-based retrospective study of all confirmed cases of melioidosis extracted from the Melioidosis Registry set up from January 2005 in HSB, the primary melioidosis reference hospital for the Alor Setar region of Kedah. Melioidosis is a notifiable disease in Kedah, and thus all clinically suspected cases are referred from peripheral health clinics and district hospitals to HSB for further clinical examination and case confirmation by culture and serology. Culture of blood specimens for confirming melioidosis in HSB is done using the BACTEC 9240 Instrumented Blood Culture System (Fluorescent series, Beckton Dickinson). All other specimens are cultured in blood Agar and MaConkey's medium. The organism (*B. pseudomallei*) is identified using the API - 20 (NE) screening system. Serology was based on the highly specific and sensitive Indirect Flourescent Antibody (IFA) test for detecting *B. pseudomallei *[14]. All data related to diagnosis, clinical presentation, treatment and associated outcomes from melioidosis confirmed patients in HSB are routinely collated into the Melioidosis Registry. The numbers of confirmed melioidosis cases from January 2005 to August 2008 were used in this study.

The predicted Alor Setar population for 2009 (223,199), obtained from the World Gazetteer database http://world-gazetteer.com, was used in the calculation of contemporary incidence in this study region (using the numbers of cases averaged over the study period from 2005 to 2008). Since data on the age-distribution of the population of Alor Setar were not available, we applied the general Malaysia age profile (US Census Bureau, International Data Base: http://www.census.gov/) to the predicted Alor Setar overall population in order derive the approximate age-risk populations for carrying out analyses of age-dependencies in the incidence of the disease in this region. Average monthly rainfall data for Alor Setar was calculated using data available on this variable for 2005 to 2008 with the Jabatan Meteorologi, Malaysia.

### Statistical analyses

Fisher's exact test or χ^2 ^test, as appropriate, was used for testing associations between categorical patient characteristics and disease outcomes. *t *tests or Analysis of Variance was used for testing differences in mean values of continuous patient variables. Poisson regressions (using age-specific population sizes as offsets) were used to model and test for age-dependencies in the incidences of disease cases. Generalized least squares (GLS) regression models were used for modeling the dependency of melioidosis cases and deaths on mean monthly rainfall and effects of months themselves. Models with no temporal correlation were compared with those incorporating temporal autocorrelation in the two *y *responses (cases and deaths) to account for any violation of independence in the residual errors [15].

## Results

### Incidence and mortality rates

**Figure 1:**
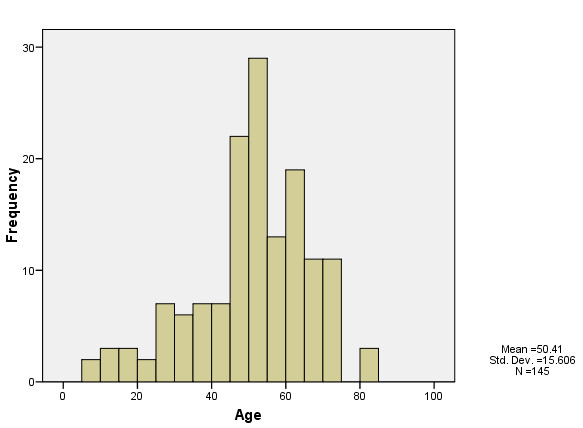
**Histogram of the frequency of melioidosis cases by host age in the Alor Setar patient population**.

A total of 145 cases of melioidosis were recorded during the 4 year period. The mean age of patients was 50.40 years (range: 7-83 years) (Table 1). Only eight patients (5.5%) were children aged < 15 years. The age-specific distribution of observed cases was distinctly non-linear (Figure 1). A poisson regression model predicting melioidosis cases using a quadratic function of age gave a good fit to the data (χ^2 ^= 102.84, df = 2, *P *< 0.0001). The majority of cases occurred among the 45 to 65 age groups before cases declined among the oldest individuals (Figure 1). Males (109 patients; 75.2%; *P *< 0.0001) predominated and the overwhelming majority of cases irrespective of gender were Malays (129 patients, 88.9%) (Table 1). There was no age difference between male and female patients overall or in relation to race. There were 49 deaths (33.8%) with no statistical difference between the mortality rates observed in male and female patients (χ^2 ^= 0.77, df = 1, *P *= 0.0.423). The ethnicity of patients also did not affect the observed mortality rates either overall or by gender. Patient occupation appeared to play a significant role in disease acquisition, with the proportion of observed cases higher among patients involved in farming, forestry and fishing, the unemployed and among the unspecified "Other" category (χ^2 ^= 30.57, df = 7, *P *< 0.0001). Mortality from the disease was also observed to be proportionately higher among these patients (χ^2 ^= 44.55, df = 7, *P *< 0.0001). Malays comprised 90.50% of patients belonging to these three occupation groups. The calculated annual incidence of melioidosis in the study region for the period from 2005 to 2008 was 16.35 per 100,000 population per year.

**Table 1:** Demographics, occupation and outcomes of 145 cases of melioidosis

	Number (% of total)	Age mean (range)	Mortality Number (% of total)
Male	109 (75.2)	51.26 (9.0-83.0)	39 (35.8)
Ethnic group			
Malay	98 (67.6)	51.52 (10.0-83.0)	36 (36.7)
Chinese	5 (3.4)	58.4 (32.0-81.0)	3 (60.0)
Indian	6 (4.1)	440.98 (9.0-70.0)	0
Others	0	-	-

Female	36 (24.8)	47.83 (7.0-72.0)	10 (27.8)
Ethnic group			
Malay	31 (21.4)	48.35 (7.0-72.0)	9 (29.3)
Chinese	4 (2.8)	48.75 (38.0-61.0)	1 (25.0)
Indian	0	-	-
Others	1 (0.7)	28.00	0

Occupation			
Administrative and Managerial	10 (6.9)	47.7 (23-62)	3 (6.1)
Farming, forestry, fishing	27 (18.6)	51.9 (29 - 66)	6 (22.2)
Industrial workers	8 (5.5)	47.2 (28-64)	1 (2.2)
Transportation work	8 (5.5)	52.6 (34-69)	3 (6.1)
Housewife	14 (9.6)	48.4 (23-72)	3 (6.1)
Student/children	10 (7.0)	18.0 (7-40)	2 (4.0)
Unemployed	27 (18.6)	59.8 (31-83)	12 (24.5)
Others	22 (15.2)	50.8 (17 - 81)	19 (38.7)

Total	145	50.40 (7-83)	49 (33.8)

### Primary diagnoses

Table 2 shows the primary clinical presentations and outcomes of the patients. Overall, 62.75% (91 out of 145) of the cases were culture positive. The overall mortality rate in the culture positive cases was 45.05% compared to only 14.8% in culture negative but sero-positive cases (OR= 4.66; 95% CL: 1.90 - 12.77; *P *< 0.001). Pneumonia accounted for 42.06% of primary clinical presentations (61 cases), with soft tissue abscess being the second major presentation (17.24% of total cases). In the culture positive pneumonia cases, the mortality rate was 64.10%, while it was 18.18% in culture negative pneumonia cases (OR = 3.48; 95% CL: 1.01 - 15.54, *P *= 0.045) (Table 2). However, although sample sizes are small, a similar 42.11% and 50.0% of patients diagnosed with soft tissue abscess in either the culture positive or the culture negative group died from melioidosis (OR = 0.85; CL: 0.13 -6.53, *P *= 0.99). Osteomyelitis/septic arthritis were also good predictors of deaths in the culture positive group (42.1% mortality), as was liver and splenic abscess diagnosed in this group (16.1% and 11.1% predicted deaths in culture positive groups although sample sizes were again small). Brain and neurological abscess appear to be equally good predictors of death in both groups (Table 2).

**Table 2:** Primary clinical presentations and outcomes of 145 cases of melioidosis

Type of primary clinical presentation^a^	Culture + ve (91 cases)		Culture - ve/Serology +ve (54 cases)	
	
	Number of patients (n)	Number died (% of n)	Number of patients (n1)	Number died (% of n1)
Pneumonia	39	25 (64.1)	22	4 (18.2)
Soft tissue abscess	19	8 (42.1)	6	3 (50.0)
Osteomyelitis/septic arthritis	7	3 (42.9)	0	-
Prostatic abscess	1	0	0	-
Liver abscess	6	1 (16.7)	4	0
Splenic abscess	9	1 (11.1)	6	0
Brain/neurological abscess	2	1 (50.0)	5	2 (40.0)
Others	40	20 (50.0)	14	3 (37.2)
Total	91	41 (45.1)	54	8 (14.8)

^a ^In some cases, a single patient had more than one presentation

Table 3 indicates that out of the 91 culture positive cases, a large number (75 patients representing 83.0% of total culture positives) were bacteremic (ie. had bacteria in blood cultures). The recorded mortality rate among bacteremics (48.0%) was significantly higher than among non-bacteremics (18.7%) (OR = 4.00; CL: 1.05 -15.19, *P *<0.05). The primarily diagnosed diseases among bacteremic patients and associated mortality rates essentially followed those given for the culture positives in Table 2.

**Table 3:** Primary clinical presentations and outcomes of 91 culture positive cases

Type of primary clinical presentation^a^	Bacteremic (75 cases)		Non-bacteremic (16 cases)	
	
	Number of patients (n)	Number died (% of n)	Number of patients (n1)	Number died (% of n1)
Pneumonia	37	24 (64.7)	3	1 (33.3)
Soft tissue abscess	8	5 (62.5)	11	2 (18.2)
Osteomyelitis/septic arthritis	5	3 (60.0)	2	0
Prostatic abscess	1	0	-	-
Liver abscess	5	1 (20.0)	1	0
Splenic abscess	7	1 (14.3)	2	0
Brain/neurological abscess	2	1 (50.0)	-	-
Others	32	16 (50.0)	6	3 (50.0)
Total	75	36 (48.0)	16	3 (18.7)

^a ^In some cases, a single patient had more than one presentation

### Associated diseases

Diabetes mellitus constituted the major underlying risk factor for developing and dying from melioidosis in this study population (Table 4). Thus, 57% of all primarily diagnosed melioidosis cases (whether culture positive or not) were positive for diabetes and this number was proportionately even higher among the culture positive cases (72%). There were accordingly significantly more patients who were positive for diabetes and melioidosis among the culture-positive group (χ^2 ^= 8.01, df = 1, *P *< 0.005), but not when all cases are considered (χ^2 ^= 2.49, df = 1, *P *= 0.115). Patients with diabetes also suffered significantly more mortality from melioidosis compared to those who did not have this risk factor (rates of 41.46% versus 23.81% respectively, OR = 2.26, 95% CL: 1.04 - 5.06, *P *= 0.033). A poisson regression of the dependency of the number of diabetic patients with age, using the number of melioidosis cases as the exposed population in each age group, indicated a significant quadratic influence of age on the diabetes cases observed in this patient population (χ^2 ^= 6.29, df = 2, *P *= 0.04). Although mortality was also high for the other disease risk factors, they occurred in a very few number of patients in this study community to assume statistical importance (Table 4).

**Table 4:** Melioidosis risk factors and mortality by risk factors

Risk Factors	Number (% total cases)	Culture positives (% with risk factor)	Died (% mortality)	p-value^a^
Diabetes mellitus	82 (56.6)	59 (71.9)	34 (41.5)	0.033
Chronic renal failure	14 (9.7)	9 (64.3)	7 (50.0)	ns
Chronic lung disease	4 (2.8)	3 (75.0)	2 (50.0)	ns
HIV/AIDS	4 (2.8)	2 (50.0)	2 (50.0)	ns
Other immuno- compromised state	5 (3.5)	4 (80.0)	1 (20.0)	ns
Others^b^	32 (22.1)	22 (68.6)	11 (34.4)	ns

^a ^Risk factor present versus absent in culture positives

^b ^No risk factors recorded for these patients

Ns, not significant

### Climatic Factors

**Figure 2:**
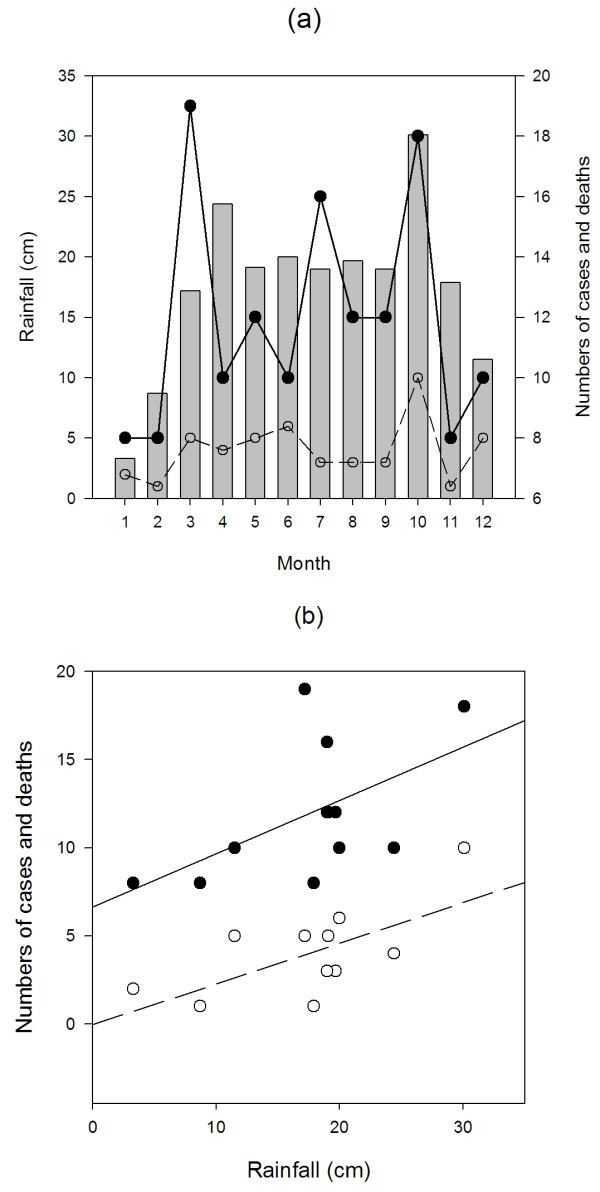
**Association of numbers of melioidosis cases and deaths in relation to (a) month and (b) total monthly rainfall (cm) in the Alor Setar region **. Columns indicate mean monthly rainfall while points indicate number of either melioidosis cases (closed circles) or deaths (open circles). Lines in (b) portray predictions of the GLS models described in the text indicating the associations of cases (solid line) and deaths (dashed line) with mean monthly rainfall respectively.

Figure 2 depicts the associations between the occurrence of cases and deaths from the disease in relation to the observed mean monthly rainfall in the Alor Setar region. The plots demonstrate that disease cases and deaths were highest in the wettest months (Figure 2a), and increased linearly with the mean monthly rainfall received in the study region (GLS model testing the dependence of cases and deaths on month and rainfall indicated a significant linear dependence with rainfall but no trend with month *per se *in both cases (*t*= 2.41, df = 9, *P *< 0.05 for cases, and *t*= 2.35, df = 9, *P *< 0.05 for deaths respectively; Figure 2b). Incorporation of residual temporal autocorrelation into this model did not improve the obtained fits.

## Discussion

The incidence of melioidosis recorded for Alor Setar (16.35 per 100,000 per year) is much higher than the rates recorded in other sites within the South East Asian region (Table 5). It is, however, comparable to the incidence of 19.6 per 100,000 per year observed previously for the Top End endemic region of the Northern Territory of Australia [16]. While high rainfall could be a common environmental factor that might underlie the high incidences observed in our study area and the Australian site, it is instructive to note that other risk factors, particularly excess alcoholism (known to be low in our study region but found to be very high in the Australian site [16]) and levels of diabetes mellitus varied too markedly between the two regions to suggest that a similarity in risk factors alone may explain the comparatively higher disease incidences in these sites. It is also unlikely that rainfall patterns and other key risk factors (Table 5), including agricultural activities, differed markedly between Alor Setar and the other South East Asian sites to account for the striking difference in infection incidence rates observed between these sites. While on the one hand this points to the need for undertaking more detailed studies comparing the epidemiological and environmental risk factors underlying the natural history of the disease using standardized data from different sites, it is notable that both the Top End and our studies are based on better notification, ascertainment and reporting of disease cases. At the very least, this suggests that the calculated mean annual incidence of 16.35/100,000 is likely to be reflective of the true melioidosis incidence in our study region. It also indicates that previous estimates of melioidosis incidence in the South East Asian region (Table 5) could be significant underestimates [12,16,17].

**Table 5:** Comparative epidemiology of Melioidosis

Variable	Australia^1^	Thailand^2^	Singapore^3^	Pahang, Malaysia^4^	This study
No. of cases	252	423	372	135	145

Incidence*	19.6	4.4	1.7	6.1	16.4
Median age (year)	49	45	55	51	50
Male:female ratio	3:1	1.4:1	4.5:1	3.6:1	3:1
Bacteremics (%)	46	60	39	92	52

Mortality rate (%)					
Overall	19	44	40	54	34
Bacteremic cases	37	-	55	54	50
Diabetes mellitus	37	20	57	74	57

^1 ^Currie et al. 2004 [16]; ^2 ^Suputtamonkol et al. 1999 [20]; ^3 ^Heng et al. 1998 [23]; ^4 ^How et al. 2005 [12]

*per 100,000 population per year

The high overall fatality rate of 33.8% (increasing to 48.0% for bacteremics) among the patients in our study confirms the deadly nature of this disease. This high observed mortality rate, despite the use of recommended standard antibacterial agents for therapy (including ceftazidime, trimethoprim/sulfamethoxazole and doxycycline), highlights the difficulty of curing melioidosis infection once it is established. Such pathogen persistence in the face of treatment may be a reflection of the ability of the organism to survive in abscesses, cells, and even in biofilm, possibly via development of *in vivo *resistance to antibiotics [6,18,19]. However, this explanation may not fully account for the observed pathogen persistence in our study, as most strains identified in our patients were found to be susceptible to the recommended antibacterial agents used in therapy (100% for ceftazidime to 82.4% for tetracycline). Our fatality finding thus raises questions regarding regional factors in disease-induced mortality. Variations in the presenting and associated disease mix among diseased populations could be a major cause of the observed between-region heterogeneity in mortality, but other factors may also play a role. These may include geographic strain differences in the pathogenicity and virulence of *B. pseudomallei *[3,6,19], host genetic differences in contracting melioidosis [20,21], and variations in both pathogen and host responses to standard treatment regimens [7]. On the other hand, it is also possible that the apparently higher relative mortality rates observed particularly in the other South East Asian settings (compared to the Top End setting of Australia) could merely be an artifact of underestimating the incidence of the disease in these areas (Table 5).

However, one major direct difference between the South East Asian and the Australian patients that could explain the regional variation in mortality rates is the higher proportion of bacteremic cases found among patients in South East Asia (Table 5). The median ages of the diseased populations across the two regions appear to be similar, and thus variation in durations of population exposure is unlikely to govern this observed difference in mortality. This suggests that the higher bacteremic cases observed among South East Asian melioidosis patients may be more a reflection of either a higher level of exposure or load of bacteria. Conversely, since these populations also have proportionately higher numbers of diabetics, and the polymorphonuclear leukocycte function (PMNL), known to be important in killing *B. pseudomallei *[22], in these patients can be impaired, it is possible that the latter risk factor (diabetes) may be more important in underlying both the higher bacteremia and mortality of melioidosis patients in South East Asian populations.

This study has shown that males in the Alor Setar region are at particular risk of acquiring melioidosis infection, supporting previous observations regarding heightened male susceptibility to the disease from other localities (Table 5). This result clearly reflects the importance of occupational exposure, since activities related to higher contact with soil (farming, forestry and fishing, and recreational or small-scale foodstuff gardening undertaken by most of the unemployed in this study) tend also to be carried out predominantly by males in this Kedah community.

Our analysis of age-specific patterns in the occurrence of melioidosis cases has shown a non-linear rise and fall of cases with patient age. Although previous epidemiological studies [17,23] have also remarked on the age-specificity of melioidosis cases or incidence in their study populations, this is first study to systematically analyze the form and the factors that may underlie any observed pattern. Our chief contribution in this respect is that the observed non-linear age-pattern in melioidosis frequency in our patients (Figure 1) may be directly related to a parallel age-dependency in the occurrence of diabetes in this population. The importance of this finding is that any acquired immunity developed by the host to *B. pseudomallei *[24] is unlikely to be significant enough to shape the epidemiology of infection in endemic populations. Instead, we suggest that the most plausible cause for the observed age-dependency in infection could be a cohort effect on the incidence of diabetes in this population, with younger patients showing higher melioidosis incidence compared to older patients as an artifact of diabetes becoming a growing health problem only recently in Malaysia [25]. As in the case of occupation, this finding again emphasizes the complex anthropogenic mediation of this disease in our study population.

That the incidence of melioidosis varied seasonally with rainfall in our study region is unremarkable given that exposure to contaminated water is a major route of pathogen transmission, but the moderate association detected supports the idea that seasonal patterns in the case of this disease may be modified in a complex manner by poorly understood interactions between climatic, environmental and host behavioural factors which may vary between localities [26,27]. Thus, the strong seasonal correlation between melioidosis and rainfall observed in northern Australia [28] and northeast Thailand [17] as compared to the weaker correlations seen previously in Kuala Lumpur, Malaysia [27], and in this study, and the absence of correlation seen in a Singapore [23] setting, could be a function of clearly defined intensely wet and dry seasons occurring in the first two locations compared to the year-round rainfall seen in the latter locations. Extreme weather patterns may clearly accentuate while more even rainfall patterns would weaker any association, suggesting that it may be periods of high rainfall intensity, which may also increase aerolisation and inhalation of bacteria [28], rather than total rainfall that may be the more important factor governing the association between melioidosis and rainfall. However, host behaviour may play a confounding role. Thus, during the rainy season in Thailand and in our study region, rice farmers plant in submerged fields leading to increased exposure of these high-risk groups to contaminated environments whereas such defined occupationally-associated seasonal risk is unlikely to occur in the urban settings of Kuala Lumpur or Singapore. These findings indicate that it is important to consider the socio-ecological context of melioidosis if we are to make better predictions of the impact of climate variation and future change on the transmission of this disease [29-32].

The multiple clinical manifestations of melioidosis has been summarized by Cheng and Currie [7], and is supported by the present findings reported here (Table 2). As with most previous studies, pneumonia accounted for nearly half of the clinical presentation in our study population with soft tissue abscesses presenting the second most important clinical syndrome. Other syndromes were too few to make any meaningful comparison with previous findings, although one major surprise was the lack of any clear cases of renal disease in our study population.

The importance of diabetes as the most commonly associated concomitant disease risk factor for melioidosis is similarly well documented [7], and the rates of this disease among our patients (57% for all patients rising to 72% among culture-positive cases) compare well with the high rates obtained from other population settings (Table 5). Given that the WHO has estimated that in the year 2030 there will be 2.48 million people with diabetes in Malaysia given current population growth rates [25], and it currently costs between US$494 to US$1,490 to treat a melioidosis patient in Kedah, it is apparent that if this expected rise in diabetes incidence goes unchecked, melioidosis will emerge as a major infectious disease in this state. Further studies into the epidemiology of this chronic-infectious disease interaction are now plainly warranted to examine this hypothesis [33].

## Conclusions

We have shown that melioidosis is a major socio-ecological infectious disease in Kedah resulting in very high mortality despite antibiotic therapy. The burden of the disease will very likely increase over the near to medium future in this region, unless better preventive measures are put into place among high-risk groups, such as rice farmers, and better interventions against associated diseases that enhance susceptibility to infection or death, such as diabetes or pneumonia and abscesses, are implemented in these populations. However, given that myriad social and ecological factors appear to govern the transmission of this disease in endemic communities, it is clear that new understanding and development of robust interventions will materialize only by recognizing and framing melioidosis as a complex transdisciplinary problem requiring the taking of an integrated approach that successfully combines the social, cultural, ecological, environmental and biomedical sciences [30-32,34].

## Competing interests

The authors declare that they have no competing interests.

## Authors' contributions

MRAH conceived and directed the study. He was the chief physician responsible for case management and also in charge of the Melioidosis Registry. NPP and NAA were involved in the diagnosis and management of the study cases. MRAH, SPP, NV, RM and EM designed the analysis and EM and KV analyzed the data. EM wrote the manuscript. All authors read and approved the final version of the manuscript.

## Pre-publication history

The pre-publication history for this paper can be accessed here:

http://www.biomedcentral.com/1471-2334/10/302/prepub
